# Monthly Summary

**Published:** 1873-12

**Authors:** 


### MONTHLY SUMMARY.

---

*Tempering Steel.*—Since, in tempering steel, the colors owe their appearance to the formation of an exceedingly thin superficial skin of oxide, it is evident that the steel, when withdrawn from the fire, does not retain its first color, but there appears other colors in consequeuce of a subsequent oxidation by the air, untill the steel is sufficiently cool. Of a certain color, one can only judge with certainty by examining the conditions under which it occurs. If two pieces of steel are heated until the yellow color appears, and if one is withdrawn, it may become in the air purple, violet, and finally blue, while the other piece assumes the same colors in the fire. However, if both pieces when blue are dipped into water, they assume different degrees of hardness, *i. e.*, the one which turned blue in the air will be harder than the one left in the fire. Hence it follows that proper caution must be observed in this respect, and steel must either be cooled rapidly, when the right color appears in the fire, or it must be withdrawn at a preceding color, if the color is to appear by after annealing. Sometimes it is intended by the process of annealing to give to articles an attractive appearance, and at the same time to prevent them from rusting by the formation of a layer of oxide. In such a case, uniform quality and heating is required.—*Druggists' Circular.*

---

*Removing a Foreign Body from the Nose.*—Accidentally opening an old number of "Ranking's Abstract," I read an article headed, "A Novel Mode of Removing a Foreign Body from the Nose," in which is related the case of a child from whose nose surgeons failed to remove a cherry-stone, and were outdone by the village barber, who administerd an emetic, and, at the moment when vomiting was about to commence, clapped a handkerchief tightly over the mouth of the child. I was reminded of the source from which was obtained a procedure I have invariably instituted in such cases, and never without success. Very many years ago, that best of practitioners, Dr. J. P. Evans, (then residing in Arkansas,) when on a visit to his native place Tazewell, Tennessee, was called to the country to see a child with a foreign substance in its nostril, which had held its position in spite of efforts for its removal directed by the professional skill of "all the region round." On the way, the Doctor was saluted by an aged negro woman, who asked him if he was going to see that child. On receiving an affirmative answer, she said: "Put yer finger 'long side the nose, tother side from the thing, and with yer own mouf over the child's mouf, blow hard, and it's bound to come out." He followed her directions, and occasioned the result as she had predicted.—*Atlanta Medical and Surgical Journal.*

---

*Treatment of Chilblains.*—"F. Rhien racommends an aqueous solution of iodine and tannin as a remedy for chilblains. He says that the result exceeded his expectations—five applications of the remedy being successfull. The application has also been tried by others, with good results when properly applied. The solution is made as follews: About an ounce of tannin is dissolAed in halt pint of water ; seventy-four grains of iodine are dissolved in an ounce and three-fourths of spirits of wine ; the two solutions are then mixed ; and enough water is added to make up the whole to two and a half pints. The remedy is applied once daily—the best time being before going to bed. The mixture is gently warmed over a very slow fire ; the affected part (*e. g.*, the hand) is dipped in it while still cold, and held there until the liquid, on being stirred, feels uncomfortably hot. The vessel is then removed from the fire, and the hand is dried over it, without gloves. The vessel used must be of earthenware or porcelain, not of metal. Care should be taken not to use too great a quantity of iodine, especially when abrasions are present. According to Rhien, four or five applications are sufficient."—*British Med. Joururnal.*

---

*Nasal Calculus.*—The following case of nasal calculus is reported by Dr. K. K. Mitter, in the *Press aud Circular* :—

On the morning of the 12th of February, 1873, a girl æt. about five years, was brought to the dispensary by her mother, who stated she had for a long time noticed something in the right nostril of the girl, which impeded her respiration to a slight extent, and which she was very anxious to have extracted.

On making an examination I saw a small oval body in the right nostril, which I thought to be a polypus. On touching it with a probe it was felt to be a hard substance. As the girl became very restless and commenced to cry, I put her under the influence of chloroform. On examining it more minutely, it was found to be a calcareous body firmly adherent to the mucous membrane of the right ala of the nose. It was extracted with a pair of small forceps after dragging it close to the orifice of the nostril by the end of a probe bent in the shape of a hook. It was followed by a gush of bleeding, which was stopped by injection with a strong solution of alum.

The body extracted was found to be a small oval calculus with a distinct hour-glass contraction in the middle.—*Med. and Surg. Reporter.*

---

*Female Professional Education.*—The new law admitting female students to the full rights of the Zurich University, has been recommended by the Cantonal Government for adoption, and the popular vote has been taken on it. Zurich seems to be considerably in advance of the rest of the world in this matter of female education, for the number of lady students has steadily increased since the courses were first opened to them informally, six years since ; and there are reported to be one hundred and nineteen of these "girl-graduates" who have actually matriculated under the existing university rules which it is proposed to egalize.—*Med. and Surg. Reporter.*

*Deep Injection of Chloroform to Tic Douloureux.*—Prof. Roberts Baraholow, in the *Clinic*, describes his method of introducing chloroform into the sub-cutaneous cellular tissue, in facial neuralgia. Though considerable pain is produced, yet the pain quickly subsides, no inflammation or abscess ensues, and the neuralgic suffering is instantly relieved. The method is thus described :

The needle is inserted under the upper lip, which is raised, and passed deeply so that its point shall rest near the infraorbital foramen The chloroform is then slowly injected. When the needle is withdrawn, firm pressure from the cheek is made over the point of insertion of the needle, and is maintained for a time to insure the diffusion of the chloroform. At the moment of injection some burning pain is experienced, but this is quickly followed by a feeling of numbness of the upper lip and anæsthesia of the parts into which the chloroform is diffused. In a very short time a distinct swelling is perceived at the site of the injection, which is at first puffy, but afterwards becomes harder and may continue indurated and swollen for a day or two. In a few seconds after the chloroform is inserted decided giddiness is experienced and the gait is staggering. Generally the giddiness passes off in a few minutes, but in one case the patient complained of an intoxicated feeling for two days. Drowsiness has usually occurred ; and thus far not decided narcosis in any case although as much as thirty minims has been administered at a dose.—*Pacific Med. and Surg. Journal.*

---

*Atropia and Salivation.*—Dr. Wilhelm Ebstein (Breslau) concludes a paper on this subject in the *Berlin Med. Wochenschrift*, as follows :

A work by my friend, Paul Grützner, in Pflüger's *Archiv*, furnishes the proof that irritation of the medulla oblongata is followed by a marked increase of salivary secretion, which is checked on the side in which the chorda tympani and sympathetic have been divided ; on the other hand, it continues on the other side, where both or one of these nerves remains intact ; so that the assumption of a salivary centre in the spinal cord is justifiable. * * *

Of far greater practical importance is the therapeutic value of atropia in the treatment of salivation as experimental investigations have so clearly indicated. We have, in atropia, a means which markedly relieves the distress of salivation. When I give my patient a hypodermic injection of 0.0016 atropia, I assure him a perfect night's rest, which would be else impossible on his constantly saturated pillow. *In salivation, atropia is the proper narcotic.*—*Clinic.*

*The Restoration of Chloroform Injured by Age.*—Where changes are induced in chloroform by time, light, moisture or atmospheric pressure, E. B Shuttleworth is of opinion (*Canadian Pharm. Jour.*) that by far the most injurious products of decomposition are chlorine and hydrochloric acid. These products may often be found in an aqueous layer floating on the top of imperfectly rectified chloroform which has been kept some time. The incomplete removal of sulphuric acid which has been used in rectification, also leads to the same change. For the restoration of chloroform that has thus become spoiled, he recommends that it be shaken with a dilute solution of hyposulphite of soda. It should then be separated from the supernatant liquid and again washed; this time with pure water. After being separated, the chloroform should be passed through filtering paper to rid it from traces of moisture, when it will be found much improved and comparatively sweet.—*Med. & Surg. Report.*

---

*A Dentist fined for giving Chloroform.*—[*The Clinic*; from the *France Medicale*, April 23, 1873.]—M. Debaralle, dentist at Lille, was brought up before the *audience correctionelle* for having given chloroform without the diploma of doctor of medicine or officer of health, and for having through "*imprudence, negligence, maladresse et inobservation des reglements*" committed an involuntary homicide by chloroform upon the person of a woman named Caron.

The tribune decided that the administration of chloroform without a diploma was an illegal practice of medicine, and that the defendant was guilty of homicide. As he was guilty of two offences, he was fined 15 francs for the first, and for the second 500 francs, with imprisonment for one month.—*Med. Times.*

---

*A Pre-Historic Skull.*—The Osage Mission [Kansas] *Journal* gives an account of a late discovery near the western line of the county. It is a human skull imbedded in a rock. and was brought to light by blasting. Dr. J. C. Weibley, of Osage Mission, thus describes it:—

"It is that of the cranium of the human species, of large size, imbedded in conglomerate rock of the tertiary class, and found several feet beneath the surface. Parts of the frontal, parietal and occipital bones were carried away by explosion. The piece of rock holding the remains weighs some 40 or fifty pounds, with many impressions of marine shells, and through it there runs a vein of quartz, or within the cranium crystallized organic matter; and by the aid of a microscope presents a beautiful appearance."—*Med. & Surg. Reporter.*

*Chloral for Toothache.*—Dr. Page, in the *British Medical Journal*, recommends chloral hydrate as a local application in cases of toothache A few grains of the solid hydrate introduced into the cavity of the tooth upon the point of a quill speedily dissolves there; and in the course of a few minutes, during which a not unpleasant warm sensation is experienced, the pain is either deadened, or more often effectually allayed. A second or third application may be resorted to, if necessary.—*Druggist's Circular and Chemical Gazette.*

Various anodynes will answer the same purpose. Among others, iodoform in one grain doses is a very efficient remedy for dental and facial neuralgia.—*Medical Cosmos.*

